# Heterologous influenza vRNA segments with identical non-coding sequences stimulate viral RNA replication in *trans*

**DOI:** 10.1186/1743-422X-5-2

**Published:** 2008-01-11

**Authors:** Stella SF Ng, Olive TW Li, Timothy KW Cheung, J S Malik Peiris, Leo LM Poon

**Affiliations:** 1 State Key Laboratory of Emerging Infectious Diseases, Department of Microbiology, The University of Hong Kong, Hong Kong SAR, China

## Abstract

The initiation of transcription and replication of influenza A virus requires the 5' and 3' ends of vRNA. Here, the role of segment-specific non-coding sequences of influenza A virus on viral RNA synthesis was studied. Recombinant viruses, with the nonstructural protein (NS) segment-specific non-coding sequences replaced by the corresponding sequences of the neuraminidase (NA) segment, were characterized. The NS and NA vRNA levels in cells infected with these mutants were much higher than those of the wild type, whereas the NS and NA mRNA levels of the mutants were comparable to the wild-type levels. By contrast, the PB2 vRNA and mRNA levels of all the tested viruses were similar, indicating that vRNA with heterologous segment-specific non-coding sequences was not affected by the mutations. The observations suggested that, with the cooperation between the homologous 5' and 3'segment-specific sequences, the introduced mutations could specifically enhance the replication of NA and NS vRNA.

## Background

The genome of influenza A virus contains 8 RNA segments of negative polarity [1]. Each virion RNA (vRNA) can be used as a template for transcription and replication to generate viral mRNA and complementary RNA (cRNA), respectively. cRNA is a faithful complementary copy of vRNA and is used as a template for vRNA synthesis. By contrast, the transcription of the viral mRNA is terminated at a track of uridines (U) which is about 17 nucleotides away from the 5' end of the vRNA template [2,3] and the polymerase then starts to polyadenlyate the mRNA by reiteratively copying of the U-track [4,5]. It is generally believed that there is a control mechanism to regulate the polymerase's transcriptase and replicase activities [6]. However, recent studies have suggested an alternative hypothesis that such switching mechanism might not exist [7-10].

Sequence analyses of all the vRNA segments revealed that the first 12 and 13 nucleotides at their 3' and 5' ends are highly conserved [11]. Extensive studies on these sequences indicated that these regions are the promoter for transcription and replication. These sequences were shown to be involved in the viral polymerase binding [12-14], cap-snatching [14,15], and transcription initiation [16,17]. The 5' and 3' ends of each vRNA are partially inverted complementary and can form a corkscrew structure that is known to be critical for the above biological processes [6]. Within these conserved sequences, there is a single natural variation (U or C) at the 4^th ^residue of the 3' end [11]. Of all the vRNA segments, the polymerase segments (PB2, PB1 and PA) invariably carry a C residue at this position (C4), whereas most of the other segments contain a U residue at this position (U4). Mutagenic studies of this polymorphic site suggested that this nucleotide variation might modulate viral transcription and replication [18,19]. Adjacent to the universally conserved regions, each vRNA segment contains additional non-coding sequences at its 5' and 3' regions. The lengths and sequences of these non-coding sequences are segment specific. Growing evidences have supported the hypothesis that these sequences are parts of the viral RNA packaging signals [20-24]. In addition, disrupting the NA segment-specific sequences were shown to have effects on viral RNA synthesis [25-27], indicating these segment specific sequences might modulate viral RNA synthesis.

## Findings

**Figure 1:**
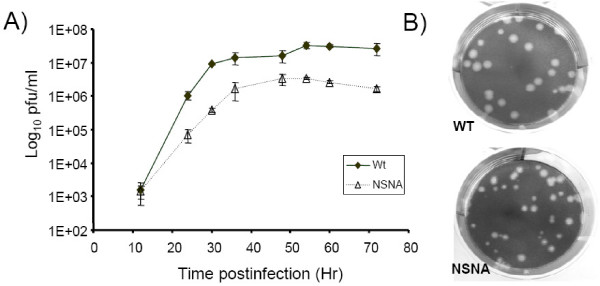
Growth properties of the wild type and NSNA mutants in MDCK cells. (A) Quantitation of infectious progeny viral particles generated from infected cells by standard plaque assays. (B) Plaque morphologies of the wild type (WT) and NSNA mutant.

In this study, we replaced the 5' and 3' NS segment-specific non-coding sequence with the corresponding sequences of the NA to investigate the role of segment-specific sequences on viral transcription and replication. An A/WSN/33 (H1N1) mutant with the above mutation (hereafter called the NSNA mutant, see Additional file 1) was generated by reverse genetics techniques [28]. The recombinant virus was titrated by standard plaque assays and the introduced mutations were confirmed by sequencing. The NSNA mutant was viable, but its maximum viral titre was about 1 log unit lower than that of the parental strain (A/WSN/33) (Fig. 1). This agreed with the previous findings that the vRNA segment-specific sequences are attenuated [26,27].

**Figure 2:**
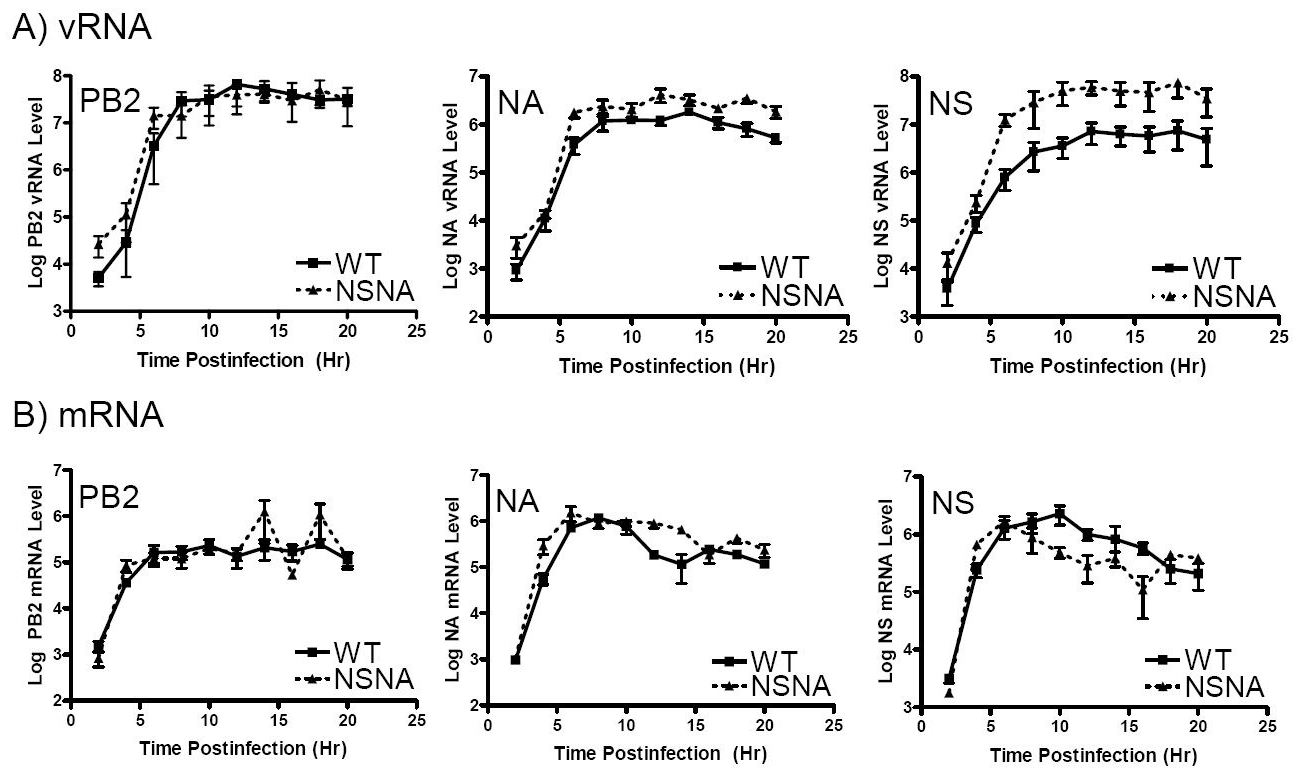
Quantitation of PB2, NA and NS vRNA (A) and mRNA (B) in cells infected with the wild-type (WT) or NSNA virus at different postinfection time points. Uni-12 primer (0.2 ng/μl) [35] was used for the cDNA synthesis of vRNA, whereas oligo dT_20 _(25 μM) was used to generated cDNA of viral mRNA. In a typical reverse transcription reaction, 0.5 μg of DNase-treated RNA sample was mixed with 1 μl of the corresponding primer, 4 μl of 5x first stand buffer, 2 μl of 0.1M dithiothreitol, and 1 μl of 10 mM deoxyribonucleoside triphosphates (Strategene), 150 U of SuperScript II reverse transcriptase in a 20 μl reaction. For detecting NA and NS RNA species, RNase-treated cDNA was examined by 5'-nuclease-based assays in a 7300 Sequence Detection System (Applied Biosystems). Briefly, 5 μl of the corresponding diluted cDNA samples were mixed with 12.5 μl superMix-UDG (Invitrogen), 0.5 μl of Rox reference dye, 1 μl of 10 mM forward primer, 1 μl of 10 mM reverse primers, 1 μl of 10 mM probe and 4 μl of water. Reactions were first incubated at 50°C for 2 min, followed by 95°C for 10 min. Reactions were then thermal-cycled for 45 cycles (95°C for 15 sec, 56°C for 1 min). Primers used in the NA detection assay were 5'-ACCGACCATGGGTGTCCTT-3' (corresponds to nt 870–888 of the NA cRNA) and 5'-GAAAATCCCTTTACTCCGTTTGC-3' (complementary to nt 998–1020 of the NA cRNA). Primer used in the NS detection assay were 5'-TACCTGCATCGCGCTACCTA-3' (corresponds to nt 277–296 of the NS cRNA) and 5'-ATGATCGCCTGGTCCATTCT-3' (complementary to nt 378–397 of the NS cRNA) were used. The probes used in the NA and NS assays were 5'-FAM-CGTCCCAAAGATGGA-NFQ-3' (corresponds to nt 950–964 of the NA cRNA; FAM, 6-carboxyfluorescein; NFQ, nonfluorescent quencher) and 5'-VIC-CACTGGTTCATGCTCA-NFQ-3' (corresponds to nt 327–342 of the NA cRNA; VIC, a proprietary dye), respectively. For the quantitation of PB2 RNA species, cDNA samples were amplified by using FastStart DNA Master SYBR Green I kit (Roche) in a LightCycler platform (Roche). In a typical reaction, 5 μl of RNase-treated cDNA was mixed with 2 μl master mixtures, 1.6 μl of MgCl_2_, 1 μl of forward primer (5'-CCGCAGTTCTGAGAGGATTC-3', corresponds to nt 2090–2109 of PB2 cRNA), 1 μl of reverse primer (5'-TCCGTTTCCGTTTCATTACC-3', complementary to nt 2226–2245 of the PB2 cRNA) and 1.6 μl of water. Reactions were first incubated at 95°C for 10 min, followed by a thermal-cycling (95°C for 10 sec, 58°C for 5 sec, 72°C for 15 sec; 40 cycles). The specificities of the amplified products were all confirmed by melting curve analysis. In all the PCR assays, serially diluted plasmids containing the corresponding sequences were used as standard controls. All the data were derived from three independent assays. The levels of mRNA and vRNA from the studied mutants were analyzed by two-tails paired t-test.

As the NA and NS vRNA of the mutant shared the identical segment-specific non-coding sequences, the effects of the mutations on the transcription and replication of these two segments were determined. Quantitative RT-PCR assays specific for the vRNA and mRNA of these segments were developed for the study. In addition, mRNA and vRNA derived from the PB2 segment were also quantitated by real-time PCR assays as controls. Total RNA from cells infected with the wild-type or the NSNA virus at an MOI of 2 was harvested at every two-hour intervals. The RNA samples were then converted into cDNA by using oligo dT_20 _or by vRNA-specific primer. The cDNA derived from the viral mRNA or vRNA was then tested by corresponding gene-specific quantitative assays (Fig. 2). As shown in the right panel of Fig. 2A, the level of NS vRNA from cells infected with the NSNA mutant was significantly higher than that of the wild type (~7.8 folds, p = 0.002). By contrast, the level of NS mRNA was slightly less than that of the wild type (Fig. 2B, right panel). Strikingly, the NA vRNA level in cells infected with the NSNA was also found to be about 2.5 folds higher than that of the wild type (Fig. 2A, middle panel; P < 0.001), but the NA mRNA levels of these viruses were statistically similar to each other (Fig. 2B, middle panel, P > 0.05). The PB2 vRNA and mRNA levels of the mutant were similar to the wild-type levels (Figs. 2A and 2B, left panels). These results suggested that the introduced mutations specifically up-regulate the replication, but not transcription, of the NA and NS segments. Interestingly, even the NS and NA vRNA expression levels are enhanced in infected cells, the NSNA mutant was shown to be attenuated. It is possible that the introduced mutations would disturb other virological processes, such as vRNA packaging [20-24].

**Figure 3:**
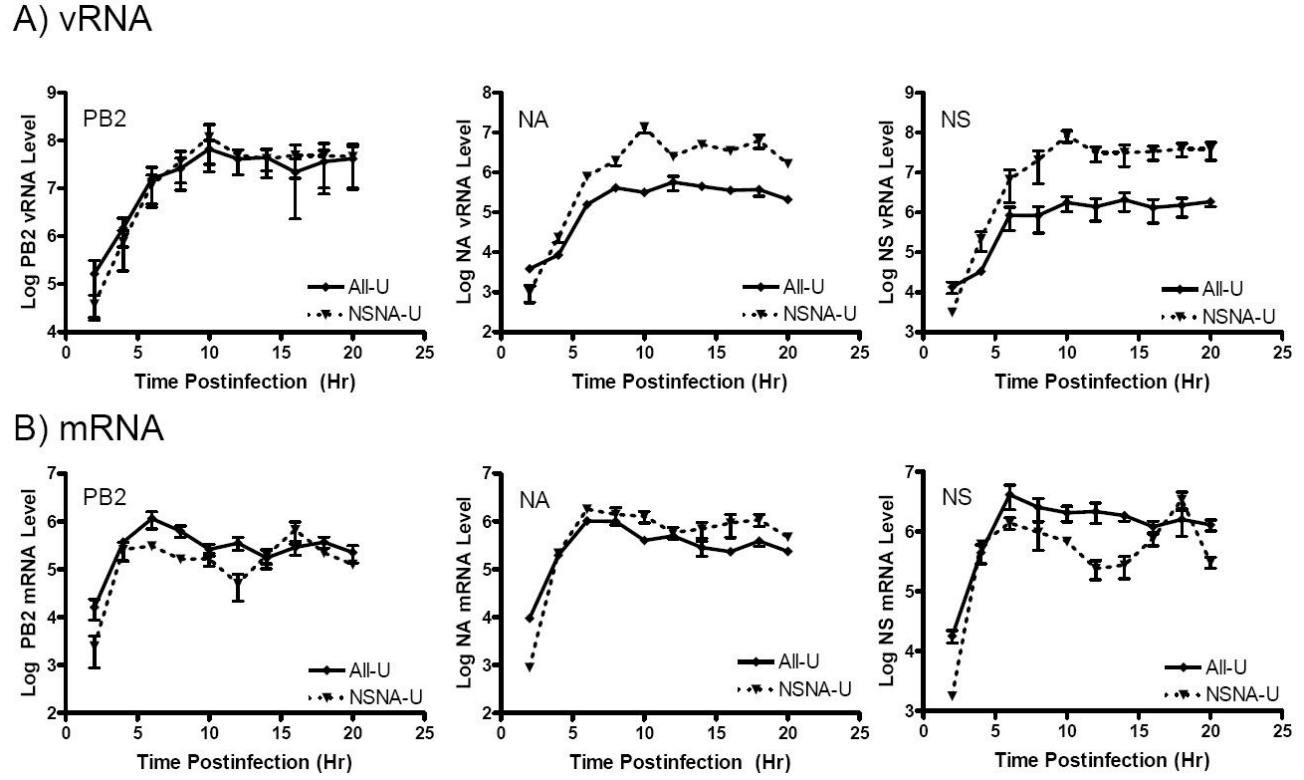
Quantitation of PB2, NA and NS RNA species in cells infected with the All-U or NSNA-U mutants at various postinfection time points. (A) PB2, NA and NS vRNA levels as indicated. (B) PB2, NA and NS mRNA levels as indicated. All the data were derived from three independent assays.

It should be noted that the 4^th ^residue at the 3' end of the PB2, PB1 and PA vRNA segments in our studied strain is a C. By contrast, all the other segments contain a U residue at this position. Previous studies indicated that sequence variations at this position would affect the viral transcription and replication [19]. To eliminate the possibility that the mutations in the NS vRNA would only affect those vRNA segments with a "U4" promoter, an additional pair of mutants was generated (Supplementary Fig. 1. All-U and NSNA-U). The All-U and NSNA-U mutants were genetically identical to the wild type and NSNA, respectively, except all the vRNA segments of these mutants contained a "C4" promoter. As shown in Fig. 3, quantitative results derived from these two mutants were similar to those observed from the wild type and NSNA mutant. Of all the analyzed RNA species, only the NS and NA vRNA levels of the NSNA-U mutants were statistically higher than those of the All-U mutant (Fig 3A, right and middle panels; P = 0.003 and 0.002, respectively). The NS and NA vRNA levels in cells infected with the NSNA-U were 14.1 and 6.7 folds, respectively, higher that those of the All-U mutant. By contrast, the NA mRNA level of NSNA-U mutant was only comparable to that of the All-U (Fig. 3B, middle panel) and the NS mRNA expression of NSNA-U was reduced (Fig. 3B, right panel). The mutations had little effects on PB2 vRNA and mRNA levels as expected (Figs. 3A and 3B, left panels). These results confirmed our observations that the mutations in NS segment could specifically up-regulate the NS and NA vRNA replications.

One of the possible mechanisms account for the elevation of NS and NA vRNA levels is that the 5' and 3' segment-specific regions would facilitate the initiation of vRNA replication. This stimulating effect, however, might require the presence of the 5' and 3' segment-specific regions from **homologous **segments. As the NA and NS vRNA segments in the NSNA and NSNA-U mutants had the identical non-coding sequences, the availability of compatible 5' ends for initiating NS and NA vRNA replications would be increased. This hypothesis is supported by two of our observations. First, our data demonstrated that the mutations had no effect on vRNA which has **heterologous** segment-specific sequences (i.e. PB2). In addition, our data showed that the transcription of the NA and NS segments were not up-regulated. These agreed with previous findings that the viral polymerase has to bind to the 5' and 3' ends of the same vRNA template for mRNA synthesis [12,29]. Thus, the increases of compatible ends' populations would not   expected to have stimulating effects on the NS and NA mRNA expressions. Interestingly, the NS mRNA levels from the NSNA and NSNA-U mutants in this study seemed to be less than that of the corresponding controls (Figs. 2B and 3B, right panels). It is possible that, due to the increase of the number of these compatible ends in infected cells, the polymerase might have less chance to bind to the ends of the same vRNA template for transcription initiation.

**Figure 4:**
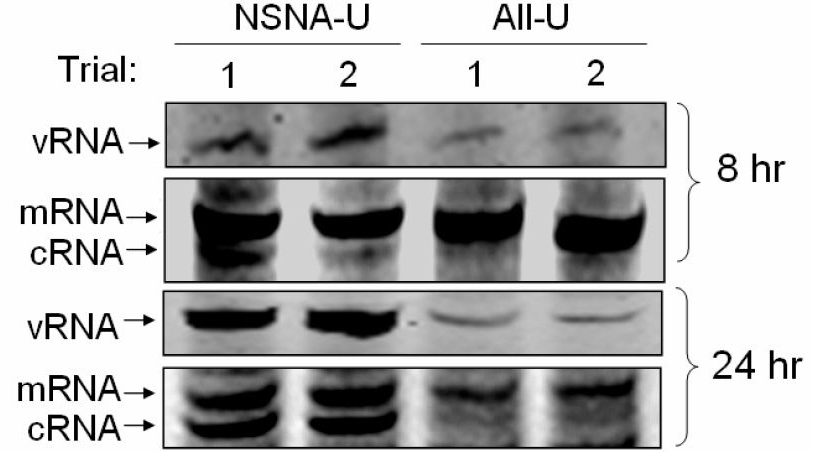
Detection of NA vRNA, cRNA and mRNA by primer extension assays. Total RNA from infected cells were harvested at 8 and 24 hr postinfection. The reaction conditions were identical to previously described assays [31], except fluorescent vRNA-specific primer (5'-Cy3-TGGACTAGTGGGAGCATCAT-3') and cRNA/mRNA-specific primer (5'-Cy5-TCCAGTATGGTTTTGATTTCCG-3') were used in the assays. The fluorescent products were resolved in 10% denaturing polyacrylamide gels and the images were analyzed by an imaging analyzer (Typhoon 8600 variable mode imager, Amersham Biosciences). Signals for the vRNA, cRNA and mRNA are shown as indicated. cRNA and vRNA signals of the NSNA-U were consistently higher than those of the All-U in independent attempts (Trials 1 and 2).

If our hypothesis was correct, the cRNA productions of the affected segments (NA and NS) were expected to be enhanced in the same fashion. To test this hypothesis, we used a primer extension assays to measure the cRNA levels in infected cells. As both NS and NA vRNA segments of the NSNA-U mutant were highly up-regulated (Fig. 3A), we used NSNA-U and All-U mutants as the studied strains in this semi-quantitative assay. Total RNA samples harvested at 8 and 24 hour post-infection were analyzed. We selected the NA segment as the target because these two mutants have the identical wild-type NA sequence. As shown in Fig. 4, cRNA levels generated from the NSNA-U were consistently higher then those of the All-U mutant. At 24 hr postinfection, cells infected with the NSNA-U had comparable mRNA and cRNA levels. By contrast, the majority of positive-stranded RNA of the All-U mutant was found to be mRNA. Agreed with our results from the quantitative RT-PCR (Fig. 3), the vRNA level of the NSNA-U was found to be much higher than the level of the All-U mutant (Fig. 4). These results further supported our findings that the 5' and the 3' segment-specific regions derived from the homologous segments might have a stimulatory effect on viral RNA replication. However, further work is required to confirm this hypothesis and we do not entirely exclude other hypotheses that might explain the above findings. For example, it is possible that the introduced mutations might also help the NS and NA vRNA form stable secondary structure in *trans*, thereby reducing the degradation rates of these vRNA [10]

In the early phase of viral infections, vRNP predominantly synthesizes mRNA for viral protein synthesis [30]. This is followed by an active phase of viral RNA replication. It was previously proposed that the nascent NP expressed in infected cells might stimulate viral RNA replication [31,32]. Recent evidences have provided an alternative hypothesis to explain this observation. Rather than stimulating the viral RNA replication, free NP and viral polymerase are proposed to protect nascent cRNA from degradation by binding to these newly synthesized cRNA transcripts [7,9,33]. The results from our current study might also help to explain the dramatic increase of cRNA levels in the late phase of viral infection. In the early phase of infection, the amount of vRNA is low and the viral polymerase is more likely to bind to the ends of the same vRNA template for transcription (i.e. activate in *cis*). Messenger RNA generated from this *cis*-acting transcription mode would be transported to cytosol for protein expression. Due to the lack of newly synthesized NP and viral polymerase, nascent cRNA generated from this *cis*-acting mode might be rapidly degraded at the early time point [7,9,33]. By contrast, during the mid- to late phase of infection, the accumulations of cRNP and vRNP make the viral polymerase complex has less chance to bind to the ends from the same vRNA or cRNA template. At this stage, the viral RNA polymerase is prone to utilize the vRNA/cRNA ends derived from different templates from transcription initiation (i.e. *trans*-activation mode). As the polyadenylation of viral mRNA requires the viral polymerase bind to the same viral template [12,29], transcription initiated by the *trans*-activation mode would favor viral RNA replication and further increase the vRNA and cRNA levels. In our study, the mutated NS segment could specifically enhance the NA vRNA and cRNA levels, suggesting the *trans*-activation mode might require the 5' and 3' vRNA ends derived from homologous RNA segments.

In conclusion, our result demonstrated that the segment specific regions have roles in controlling viral transcription and replication. Viral RNA with compatible segment-specific sequences might facilitate viral replication in *trans*. Given the fact that different viral RNA segments might have subtle sequence requirements for viral RNA synthesis [34], further studies on the segment-specific non-coding regions in other viral segments are needed.

## Competing interests

The author(s) declare that they have no competing interests.

## Authors' contributions

SSFN and TKWC generated and characterized the recombinant viruses. OTWL designed and performed the primer extension assay. JSMP analyzed the data and involved in the experimental design. LLM prepared the manuscript and participated in the design and coordination of the experiments.

## Supplementary Material

Additional file 1 NS vRNA sequences in the studied mutants. The non-coding sequences of NS vRNA in the wild-type (WT) and NSNA viruses were shown. The NS and NA segment-specific sequences were underlined and bolded, respectively. Click here for file
