# Supplementary material for: Heterologous influenza vRNA segments with identical non-coding sequences stimulate viral RNA replication in trans
Source: Virol J. 2008 Jan 11;5:2. doi: 10.1186/1743-422X-5-2 (PMC2263042; doi:10.1186/1743-422X-5-2)
Supplement: Additional file 1 — NS vRNA sequences in the studied mutants. The non-coding sequences of NS vRNA in the wild-type (WT) and NSNA viruses were shown. The NS and NA segment-specific sequences were underlined and bolded, respectively. [file 1743-422X-5-2-S1.pdf]

Viruses	NS vRNA Sequence (5' to 3')		
WT	AGUAGAAACAAGGGUGUUUUUUAUUA	NS ORF in negative sense	UAUGUCUUUGUCACCCUGCUUUUGCU
NSNA	AGUAGAAACAAGG **AGUUUUUUGAACAAA**	NS ORF in negative sense	**UUAAACU**CCUGCUUUUGCU
